# Inactivation of *Salmonella* Typhimurium, *Escherichia coli*, and *Staphylococcus aureus* in Tilapia Fillets (*Oreochromis niloticus*) with Lactic and Peracetic Acid through Fogging and Immersion

**DOI:** 10.3390/foods13101520

**Published:** 2024-05-14

**Authors:** Matheus Barp Pierozan, Jordana dos Santos Alves, Liege Dauny Horn, Priscila Alonso dos Santos, Marco Antônio Pereira da Silva, Mariana Buranelo Egea, Cíntia Minafra, Leandro Pereira Cappato, Adriano Carvalho Costa

**Affiliations:** 1 Campus Rio Verde, Instituto Federal Goiano, Rio Verde 75901-970, Brazil; matheus.pierozan@estudante.ifgoiano.edu.br (M.B.P.); jordanadossantosalves@hotmail.com (J.d.S.A.); liegedouny@gmail.com (L.D.H.); priscila.santos@ifgoiano.edu.br (P.A.d.S.); marco.silva@ifgoiano.edu.br (M.A.P.d.S.); mariana.egea@ifgoiano.edu.br (M.B.E.); adriano.costa@ifgoiano.edu.br (A.C.C.); 2 Campus Samambaia, Universidade Federal de Goiás, Goiânia 74605-450, Brazil; cintia.minafra@ufg.br

**Keywords:** aerosolization, microbial inactivation, preservatives, food safety, *Escherichia coli*, *Salmonella* Typhimurium, *Staphylococcus aureus*

## Abstract

This study investigated the antimicrobial effects of lactic acid (LA) (3%) and peracetic acid (PA) (300 ppm) on tilapia fillets (*Oreochromis niloticus*) by fogging (15 min) or by immersion (2 s) in a pool of *Escherichia coli* (NEWP 0022, ATCC 25922, and a field-isolated strain), *Staphylococcus aureus* (ATCC 25923 and a field-isolated strain), and *Salmonella* Typhimurium (ATCC 13311 and ATCC 14028), as well as the effects on the physicochemical characteristics of the fillets. Fogging was effective and the best application method to control *S*. Typhimurium regardless of the acid used, promoting reductions of 1.66 and 1.23 log CFU/g with PA and LA, respectively. Regarding *E. coli*, there were significant reductions higher than 1 log CFU/g, regardless of the treatment or acid used. For *S. aureus*, only immersion in PA showed no significant difference (*p* < 0.05). For other treatments, significant reductions of 0.98, 1.51, and 1.17 log CFU/g were observed for nebulized PA, immersion, and LA fogging, respectively. Concerning the pH of the samples, neither of the acids used differed from the control. However, treatments with LA, and fogging with PA, reduced the pH compared to immersion in PA. As for color parameters, L* and a* values showed changes regardless of the acid or method used, resulting in an improved perception of fillet quality. These results indicate that fogging and immersion are alternatives for reducing *S.* Typhimurium, *E. coli*, and *S. aureus* in tilapia fillets.

## 1. Introduction

In recent years, raw, undercooked, or lightly processed fish consumption has increased dramatically worldwide, representing a greater risk of contracting foodborne diseases [1,2]. Between 1998 and 2015, 857 fish consumption outbreaks were reported in the USA, resulting in 4815 illnesses, 359 hospitalizations, and 4 deaths [3]. Meanwhile, in Brazil, the Notifiable Diseases Information System (SINAN) recorded 60,907 cases of food poisoning across the country, resulting in 71 deaths [4]. Among the main types of fish consumed, tilapia is the third-most important fish in the world [5]. In 2019, the world production of tilapia was 4.59 million tons, representing an estimated commercial value of USD 9.1 billion [6]. In addition, tilapia is among the best choices for fish because of their characteristics and low levels of mercury [2].

Owing to their intrinsic and extrinsic factors, fish are excellent culture media favoring microbial growth [7,8] and are one of the food categories commonly implicated in foodborne outbreaks. Thus, the fishing industry has great difficulty in maintaining fish quality, often due to considerable distances between consumers and harvesting or production areas, offering opportunities for microbial growth and recontamination [9].

In addition to contamination by the natural microbiota, fish can be easily contaminated during processing by spoilage microorganisms and pathogens, such as *E. coli*, *Salmonella* spp., and *S. aureus*, which are important pathogens causing food outbreaks, including fish [3,9]. According to Barret et al. [3], *Salmonella* is among the main microorganisms that cause diseases in fish, resulting in 97 cases (31%) of hospitalizations in outbreaks between 1998 and 2015 in the USA. In this scenario, outbreaks involving *S. aureus* enterotoxin (3 cases) and Shiga toxin-producing *E. coli* (5 cases) resulted in 8 and 70 illnesses (26 hospitalizations), respectively [3].

Although these pathogens are not a part of the natural microbiota of fish, they can become asymptomatic hosts, causing cross-contamination during the industrialization and commercialization stages [10]. This cross-contamination is mainly a result of handling and the extraordinary capacity of fish for biofilm formation, representing great dangers for the food industry [11].

Techniques other than conventional processes have shown the potential to improve food safety and quality [12]. In these cases, the use of antimicrobial agents, such as organic acids, essential oils, and bacteriocins, among others, has gained significant prominence in the preservation of fish as an additional step for the safe production and shelf-life extension of these products [12,13,14,15].

The FDA recognizes organic acids as food substances; therefore, these acids are generally recognized as safe (GRAS) through FDA regulation 21CFR184.1061 [16]. As a result, organic acids, such as lactic acid and peracetic acid, are frequently used in the food industry for surface food decontamination, with a considerable emphasis on animal carcasses, fruits, and vegetables [13,17,18].

In addition to spray application and immersion, fogging, also known as aerosolization or chemical fogging, has gained prominence for the surface decontamination of foods with organic acids. The technique involves applying products in the form of a fine mist, resulting in the uniform deposition (3D) of sanitizers on the surfaces of products, which reduces costs related to the amount of product used and water consumption. In addition, fogging can be employed in the environmental decontamination of processing rooms and equipment and is a very versatile technique for safe food production [19,20].

Despite the advantages of surface decontamination using organic acids, its application in the fishing industry is rare. Additionally, reports on this topic are rare in the scientific literature, even though this technique has significant potential for the fishing sector [21]. Therefore, this study aimed to evaluate the effect of the use of organic acids (lactic and peracetic), applied via fogging and immersion, on the inactivation of pathogens (*S.* Typhimurium, *E. coli*, and *S. aureus*) in tilapia fillets, and to evaluate its effects on the physicochemical characteristics (pH, color, and texture profile) of the fillets.

## 2. Materials and Methods

### 2.1. Sample Preparation

Tilapia fillet samples were acquired from a slaughterhouse that works under the state inspection regime in the city of Quirinópolis, Goiás, Brazil (18°15′22.90″ S, 50°08′28.95″ W). Fish were captured in culture tanks close to the slaughterhouse and processed. The fillets were collected after the final packaging, before freezing, and then transported, under ice (fish/ice of 1:3), to the Laboratory of Applied Microbiology of IFGoiano—Campus Rio Verde—GO. The packaged fillets were aseptically removed and filleted in a sterile tray and cut into pieces of approximately 10 g each (6 cm long × 2 cm wide and 0.3 cm thick) aseptically in a laminar flow chamber. The fillets were then placed on sterile plates (90 mm diameter) and subjected to UV light for 10 min on each side to promote surface decontamination of the microbiota present. After this decontamination process, no such microorganisms were detected in the fillets.

### 2.2. Bacterial Strains and Inoculation Preparation

For the experiment, two strains of *Escherichia coli* (NEWP^®^ 0022, ATCC^®^ 25922™), two strains of *Staphylococcus aureus* (ATCC^®^ 25923™ and a field-isolated strain), and two strains of *S.* Typhimurium (ATCC^®^ 13311™ and ATCC^®^ 14028™) were used. *S. aureus* strain isolates were kindly provided by INTEGRALAB, a laboratory in Cascavel, PR, Brazil.

Bacterial suspensions stored in refrigeration on nutrient agar were individually activated in Brain Heart Infusion Broth (BHI, BD, Becton Dickinson and Company, Sparks, MD, USA) and incubated under aerobic conditions for 24 h at 37 °C. After this period, the tubes were centrifuged at 3500 rpm (5190× *g*) for 10 min at 4 °C, the supernatants were discarded, and the pellets were washed three times in a row using sterile saline (0.85% *w*/*v*). Then, the pellets were rehydrated in sterile saline to adjust the concentration to 10^8^ log CFU/mL per inoculum using the MacFarland scale (0.5 suspension). For the elaboration of the pool of strains of each pathogen, equal proportions of each strain were mixed, thus generating a pool for *E. coli*, *S.* Typhimurium, and *S. aureus* strains, forming three separate culture pools. The pool concentration of each strain was determined via plate counting.

### 2.3. Preparation of Tilapia Fillet Samples and Application of Surface Decontamination Techniques (Immersion and Fogging)

For each fillet, 0.1 mL of bacterial culture was inoculated from the pool of each pathogen onto the fillet surface (center of the fillet), reaching a concentration of approximately 10^6^ CFU/g. Then, the samples were left in laminar flow inside the Petri dishes for 5 min without UV light to dry/fix the aliquots on the fillet surface.

The treatment conditions (solution concentrations and processing time) were defined based on previous experiments, and no undesirable organoleptic changes were observed on the fillets (unpublished data).

The immersion and fogging times were determined through experimental testing in which the fillets were weighed before and after acid application, both during immersion and fogging. The primary purpose of these tests was to determine the optimal immersion and fogging times to ensure that the fillets absorbed comparable amounts of acidic solution, regardless of the application method or type of acid used.

During the initial phase of the experiments, it was found that immersion for two seconds resulted in an average absorption of approximately 410 milligrams of acidic solution while fogging for 15 min resulted in 407 milligrams. These results demonstrated a remarkable equivalence in absorption between the two application methods.

To carry out the experiments, 45 pieces of tilapia fillets (6 cm long × 2 cm wide and 0.3 cm thick) were divided into five equal groups, as follows: (1) positive control; (2) fogging for 15 min with 3% lactic acid; (3) immersion for 2 s with 3% lactic acid; (4) fogging for 15 min with 300 ppm peracetic acid; and (5) immersion for 2 s with 300 ppm of peracetic acid. Each treatment was replicated thrice. The organic acids tested consisted of L-lactic acid USP 40 levogyrous (88.15%) (Labsynth^®^, São Paulo, Brazil) and commercial peracetic acid (15%, Proxitane^®^ 1512 Thech^®^) (Solvay, Brussels, Belgium). All solutions were prepared with sterile distilled water.

For fogging, a domestic ultrasonic humidifier was used (Model U-04 3.7 L Verde Bivolt Nac Premium 25 W—Ventisol^®^, Palhoça, Santa Catarina, Brazil), adapted to an extension hose, to carry the fogging to the experimental box where the fillet samples were kept. Before use, the device was cleaned, sanitized with 70% alcohol, and placed under laminar flow in UV light for 15 min.

**Figure 1:**
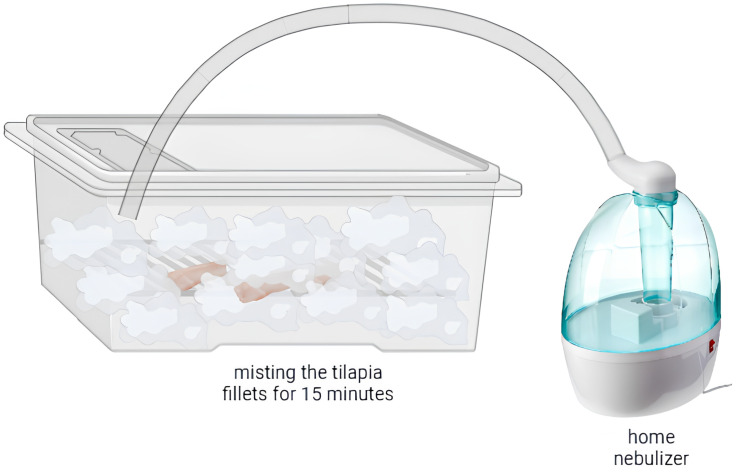
The fogging system during operation on tilapia fillets in a prototype model system. Figure was created in BioRender.com.

The inoculated fillet samples were placed on a sterilized stainless steel screen inside an acrylic box. The extension hose was inserted into the upper part of the box, allowing for the exit of the solution of each acid from the nebulizer to the fillet samples. System saturation occurred one minute after the start of the process, observed when relative humidity was >90%. Figure 1 shows the fogging system during operation on tilapia fillets in a prototype model system.

### 2.4. The Determination of the Antimicrobial Effects on Tilapia Fillets of the Use of Organic Acids through Immersion and Fogging

After applying the treatments, the samples were added to flasks containing 90 mL of Dey–Engley Neutralizing broth solution (MERCK^®^). Serial decimal dilutions were performed to count the bacterial survivors, and surface plating was performed in different media, with Hektoen enteric agar for *Salmonella* spp., Baird-Parker agar for *S. aureus*, and Violet Red Bile agar for *E. coli*. Subsequently, the plates were incubated at 36 °C for 24 ± 2 h, and the results were expressed as log CFU/g. The plating analyses were performed in duplicate.

### 2.5. Analysis of Quality Parameters

The same treatment conditions, as described above, were used to determine the physicochemical characteristics, except for the inoculation of the strains. For pH analysis, a digital benchtop pH meter was used (Luca210, Lucadema^®^, São Paulo, Brazil), with 10 g of the sample homogenized in 100 mL of distilled water. This analysis was performed in triplicate for each treatment.

Instrumental color was determined at four different points in each sample, which were analyzed in triplicate, and the average of these values was calculated. A Colorquest II benchtop spectrophotometer (Hunter Lab, ColorFlex EZ, Reston, VA, USA) with a CIELab color scale system (Commission Internacionale de L′Eclairage, Vienna, Austria) was previously calibrated for measurement in RSIN mode in CIE L*, a*, b* space.

Shear force (kgf/cm^2^) and texture profile analysis (TPA) were performed using an Ametek Texture Analyzer Brookfield CT3 (TexturePro CT V1.9 Build 35^®^, Brookfield Engineering Labs. Inc., Middleboro, MA, USA). TPA was performed by observing the instrumental texture parameters (hardness, adhesiveness, gumminess, chewiness, cohesiveness, and elasticity) [22]. Three and six cylindrical samples (30.50 mm in diameter × 15.50 mm in height) were taken from the same location on each fish fillet and subjected to two compression cycles to obtain the texture profile and shear force, respectively. For texture analysis, the samples were compressed to 30% of their original height with a cylindrical probe (TA4/1000 Cylinder 38.1 mm D) of 50.8 mm in diameter using a test speed of 2 mm/s. Shear was coupled to a Warner-Bratzler device (HDP/WBV).

### 2.6. Experimental Design—Statistical Analysis

After the exploratory analysis, an analysis of variance (ANOVA) was performed, considering the effects of additional treatment, organic acid, the application method, and the interaction between the organic acid and the application method. The Dunnett test was carried out to compare the control treatment with the other treatments, while the Scott–Knott test was carried out using the computer package “ExpDes.pt”. A multivariate factor analysis was performed using the “Mvar.pt” software (Version 2.2.1, 19 August 2023) package to obtain a global understanding of the results and to discriminate between treatments. Statistical analyses were performed using the R Development Core Team computer program (version 3.5.0, 23 August 2018).

## 3. Results and Discussion

### 3.1. The Determination of the Antimicrobial Effects on Tilapia Fillets of the Use of Organic Acids through Immersion and Fogging

**Figure 2:**
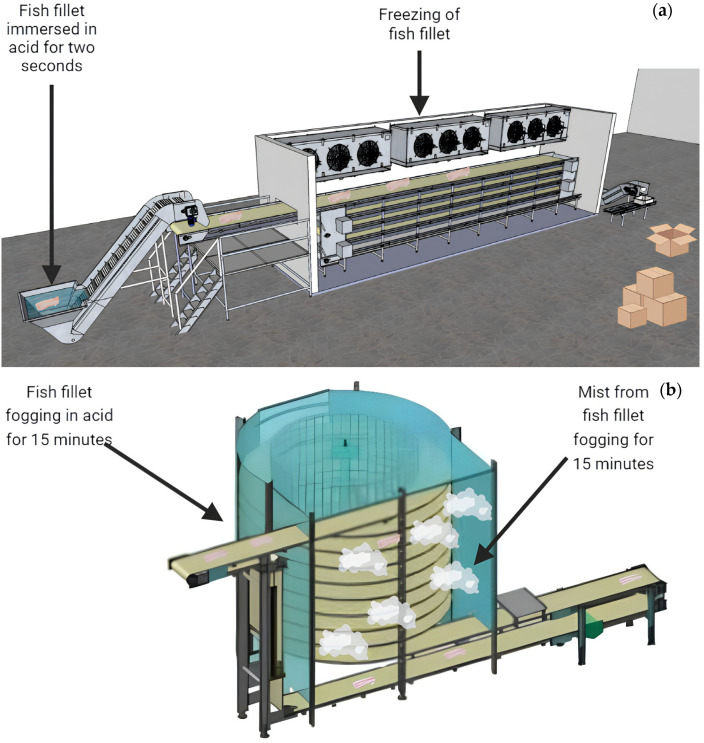
(**a**). Commercial system for freezing fish fillets after immersion in acid. (**b**). Commercial acid fogging system for 15 min prior to freezing. Figure was created in BioRender.com.

Figure 2 presents an illustration of industrial-scale immersion and nebulization systems. Table 1 presents the antimicrobial effects of using organic acids (lactic and peracetic acid) under fogging or immersion on the survival of the pool of analyzed pathogens (*S.* Typhimurium, *E. coli*, and *S. aureus*). For *S.* Typhimurium and *E. coli*, there was no significant effect from the interaction between the method and the acid. For *S. aureus*, an effect from the interaction between the method and the acid was observed.

Regarding the population of *S.* Typhimurium, there was a significant effect only from the application method. Regardless of the acid used, the 15 minute fogging process proved to be the best method for microbial reduction compared to immersion (*p* < 0.05). However, in the population of *S.* Typhimurium, the control treatment differed from the fogging treatments and did not differ from the immersion treatments, regardless of the acid used. Compared to the control, reductions of 1.66 and 1.23 log were observed for fogging with peracetic acid and lactic acid, respectively.

There was a reduction in the number of *E. coli*, both by fogging and immersion, regardless of the acid applied compared to the control. The acids, regardless of the method, did not differ from each other. All treatments resulted in a reduction of >1 log of the pathogen.

It was found that immersion in peracetic acid did not present a significant difference compared with the control for the pool of *S*. *aureus*. Lactic acid treatment and peracetic acid fogging reduced *S. aureus* compared to immersion in peracetic acid. Reductions of 0.98, 1.51, and 1.17 log were observed for nebulized peracetic acid, immersion, and lactic acid fogging, respectively. Reductions of 1 log are of great biological significance [23,24].

Despite its wide application for products of animal origin, such as the decontamination of bovine and chicken carcasses, studies evaluating the use of organic acids for the decontamination of fish are scarce in the scientific literature [21]. According to Baptista et al. [13], immersion with acids and their use in ice manufacturing are methods currently in use. However, no reports are available concerning fogging as an alternative method for microbiological inactivation in fish. Thus, because of the scarcity of studies relating to decontamination in fish, a comparison with similar research involving other species of animals, such as birds and bovine carcasses, can serve as a model for the fish industry. Through this study, we aim to promote interest and reports on the potential of these technologies for product decontamination in the fishing industry.

The results of this study demonstrated the antimicrobial effects of lactic acid immersion on *E*. *coli* and *S. aureus*, similar to the study by Metin et al. [22], in which immersion for 30 min in lactic acid solutions (0% to 4%) resulted in the shelf-life extension of *Scomber japonicus* fillets. Smyth et al. [23] were unsuccessful in the microbiological reduction in *Gadus morhua* after immersion for 30 s in solutions containing 5% lactic acid and 5% citric acid.

Lactic acid, as well as other acids (acetic, citric, ascorbic, and other organic acids), are approved or listed in FDA regulations for various technical purposes, such as for acidulants, antioxidants, flavoring agents, pH adjusters, nutrients, and preservatives. In addition, the USDA, through the Food Safety and Inspection Service (FSIS), allows the use of these acids as antimicrobial agents as processing aids to treat poultry, beef, and pork meat through washing, rinsing, soaking, misting, spraying, immersion in cold or scalding water, and for pre and post-cooling, in concentrations not exceeding 5% [24].

When comparing the effect of immersion with lactic acid in other animal species, the results were partially similar to those found in the present work. Anang et al. [25] reported a reduction of 1.97, 1.71, and 2.59 log CFU/g, respectively, for *L. monocytogenes*, *S.* Enteritidis, and *E. coli* O157: H7 in raw chicken breasts immersed (30 min) in a lactic acid solution (2%). Laury et al. [26] observed a reduction of 2.3 log CFU/mL for *Salmonella* in poultry carcasses immersed in a buffered mixture of 2.5% lactic acid and citric acid, a result not found in this study for *Salmonella* in fish.

Spoilage bacteria are commonly Gram-negative and produce odors and flavors in seafood due to their metabolic activities [27]. Table 1 shows the reductions in the Gram-negative bacteria analyzed (*S.* Typhimurium and *E. coli*). This may be related to the ability of undissociated acids (protonated forms) to cross the lipopolysaccharide membrane of Gram-negative cells [28]. Once inside the cell, the neutral or higher pH of the cytoplasm causes the acid to dissociate into anions and protons, causing an increased intracellular acidity of the cytoplasm, which alters enzymatic reactions and nutrient transport systems, resulting in cellular metabolic disorders [28].

The application of other mixed or individual acids has also shown potential antimicrobial effects in fish in other studies. El-shemy et al. [29] observed an increase in the shelf-life of up to 12 days for fillets of *Tilapia nilotica* after immersion for five minutes in an aqueous solution containing a combination of 1% acetic acid and 3% citric acid. In shrimp, Nirmal and Benjakul [30] found significant reductions (*p* < 0.05) in psychrophilic bacteria, H_2_S-producing bacteria, and Enterobacteriaceae in *Litopenaeus vannamei* after soaking in ascorbic acid (0%, 0.005%, and 0.01%) combined with 0.1% green tea extract. In addition, the authors observed reduced rates of increases in pH, nitrogen content of total volatile bases (TVB-N), and reactive thiobarbituric acid.

In addition to immersion, other methods of applying organic acids, such as spray application and use on ice, have gained prominence for fish preservation. Monirul et al. [31] applied a mixture of acetic acid (1%) and ascorbic acid (2%) via spray on *Hypophthalmichthys* fish fillets molytrix (at 4 °C) and observed better quality preservation and shelf-life compared to other treatments.

Sanjuás-Rey et al. [32] observed microbiological reductions during the refrigeration of fish (*Merluccius merluccius, Lepidorhombus whiffagonis,* and *Lophius pescatorius*) using ice containing a commercial acid mixture comprising ascorbic, citric, and lactic acid at 800 mg/kg. In another study, Sanjuás-Rey et al. [33] verified the effect of including a mixture of citric, ascorbic, and lactic acid (0.050%) as a freezing medium for *Scomber scombrus* fish, resulting in lower bacterial growth (aerobic, anaerobic, psychrotrophic, Enterobacteriaceae, lipolytic, and proteolytic) and lower contents of total volatile bases and trimethylamine. In addition to these studies, García-Soto et al. [34] applied organic acids in the ice system (1.25 g/L of citric acid and 0.50 g/L of lactic acid) to significantly retard the growth of microbial groups (aerobic mesophiles, anaerobes, proteolytic bacteria, and Enterobacteriaceae) in the muscle of fish (*Merluccius merlucius* and *Lepidorhombus whiffagonis*).

In addition to lactic acid, the results of the present study show (Table 1) that peracetic acid also has antimicrobial effects on pathogens in tilapia fillets. Peracetic acid and lactic acid are permitted for the surface decontamination of food. In meat, poultry, and eggs, for example, 50–2000 parts per million (ppm) are allowed and can be applied by several methods, including spray cabinets, dip tanks, hand spray pumps, and coolers [24]. Peracetic acid significantly affects DNA and its bases. In addition to its effect on vegetative cells, peracetic acid has a sporicidal effect owing to its ability to damage the germination receptors for nutrients on the inner membrane proteins GerB and GerK [35].

Concerning the antimicrobial activity of peracetic acid in fish, the results of the present work are similar to those of Tong Thi et al. [36] on *Pangasius hypophthalmus* fish fillets. The authors washed the fillets in a peracetic acid solution (10, 20, 50, and 150 ppm for 10, 20, and 240 s), reducing 0.4 to 1.4 log CFU/g for *E. coli.* In another experiment, the application of peracetic acid (200 ppm for 4 min) by the immersion of *Sparus aurata* fish significantly delayed the growth of spoilage microorganisms (total viable count, *Pseudomonas* spp., Enterobacteriaceae, and H_2_S-producing bacteria), increasing the shelf-life by seven days [37].

Owing to the lack of studies on the inactivation of pathogens using peracetic acid in fish, comparing our results with experiments carried out in other animal species can serve as a model to promote interest in this topic. Nagel et al. [38] observed a reduction in *S.* Typhimurium in poultry carcasses treated in immersion tanks with 0.04% and 0.1% peracetic acid. Zabot et al. [39] observed the effectiveness of lactic and peracetic acids for microbial reduction in 54 strains of *Salmonella* Enteritidis, *S.* Typhimurium, and *Salmonella* Heidelberg isolated from chicken slaughterhouses.

In addition to its application in animals, the use of peracetic acid for the inactivation of pathogens has been reported in fruits, and there is evidence of its effectiveness for surface decontamination, regardless of the food matrix. Lukasik et al. [40] found that washing strawberries with 100 ppm peracetic acid produced a 96.8% reduction in *E. coli* O157:H7 and a 97.3% reduction in *Salmonella* Montevideo.

In addition to the processing time, conditions, type of acid used, and their respective concentrations, the application method dramatically influences the success of surface decontamination. As discussed earlier, the main techniques used for fish and meat and carcasses are immersion, spraying, and ice system techniques (only for fish). However, fogging has gained prominence not only for the surface decontamination of food but also for the decontamination of surfaces and environments, proving to be a technology with great potential for the food industry due to its effectiveness and versatility. In addition, this technique makes it possible to reduce the volume of sanitizing solutions and water consumption [20].

Despite being a technology of great potential, no studies have reported using fogging as a conservation technique for fish or meat and derivatives, for which surface decontamination with acids is already well established. Fogging has been reported only for environmental and surface equipment decontamination and for fruits and vegetables [20]. Recently, nebulizing disinfectants have been studied and even used in human disinfection chambers, proving a vital alternative to combat COVID-19. Fogging can also be used in factories and processing lines [41].

The results of the present study indicate that fogging is an up-and-coming technology, significantly reducing the analyzed pathogens. Fogging was effective using either lactic or peracetic acid (Table 1). Such results are of high importance due to the innovation of this study, and our findings may represent a new field of research in fish conservation processes.

### 3.2. The Effects of the Use of Organic Acids by Immersion and Fogging on the Quality Parameters of Tilapia Fillets

The parameters of color and pH variation in the tilapia fillets after the treatments were studied and are presented in Table 2. The effect of the interaction between the method and the acid on the variables L* (luminosity) and pH was verified.

Regarding the variable L*, the treatments by immersion, regardless of the acid used, showed higher values than the other treatments. With fogging, it was found that lactic acid produced higher values than peracetic acid. This may be attributed to the higher acidity of the lactic acid solution (pH 2.01) when compared to the acidity of the peracetic acid solution (pH 3.41), which can cause greater protein denaturation, affecting brightness. All treatments increased L* values, resulting in greater whitening in tilapia fillets. This minimizes the loss of color during storage and can be explained by the inhibitory action of organic acids on the polyphenol oxidase enzyme, which is responsible for the enzymatic browning of fish [41].

Regarding the variable a* (red color intensity), all acid treatments differed from the control but did not differ from each other. Normally, a* values in tilapia fillets are reduced during storage, mainly because of the oxygenation of myoglobin, which occurs when molecular oxygen binds to myoglobin to form oxymyoglobin (MbO_2_). Meat tissue primarily containing myoglobin (deoxymyoglobin) is purple-red. After myoglobin oxygenation, the tissue color changes to the usual bright red.

Purple myoglobin and red oxymyoglobin can be oxidized, changing the state of iron from ferrous to ferric. If this change occurs by auto-oxidation, these pigments acquire the undesirable red-brown color of metmyoglobin (MMb) [42]. Therefore, the value of a* is an indicator for estimating the quality and freshness of fish fillets [43]. The color stability of fillets treated with peracetic or lactic acid, regardless of the treatment applied, was improved by increasing the values of this variable.

Acid also affected the variable b* (yellow color intensity). The b* values of tilapia fillets treated with lactic acid were higher than those treated with peracetic acid, regardless of the application method. However, the use of acids did not differ from the control. Lactic acid produced more yellowish samples, improving consumers’ perception of fillet quality. This result is similar to that found by Tong Thi et al. [36], in which fish fillets of *Pangasius hypophthalmus* washed with 50 ppm peracetic acid for 10 s and 40 s did affect b* values.

Regarding pH, neither of the acids used differed from the control. However, lactic acid treatment and peracetic acid fogging reduced the pH compared to immersion in peracetic acid. The initial pH of the fillet samples was 6.58 ± 0.03, which follows Brazilian legislation, which stipulates a pH for fish below 7.00, excluding species of the families *Gadidae* and *Merluccidae*, whose value must be a maximum of 7.20 [44]. Regardless of the acid type and treatment applied, all samples were within the pH range of 6.18 to 6.77. The pH of live fish muscle is close to 7.0. However, post-mortem pH can range from 6.0 to 7.1 depending on the season, species, stress, and other factors [45].

Our results agree with Mohamed and Ammar [46], who found that the pH of tilapia fillets immersed in lactic acid was significantly lower (*p* < 0.05) than that of the control. The lower pH values of fillet samples subjected to lactic acid can be attributed to the lower pH of the solution (pH 2.01) compared to the peracetic acid solution (pH 3.41). pH values above 7 can limit the shelf-life of certain fish species, as these increases in pH are related to the accumulation of basic substances, such as ammonia and trimethylamine (TMA), which are produced by microorganisms [47].

Concerning texture (Table 3), the interaction between the method and the acid affected only on hardness. Treatment with lactic acid and fogging with peracetic acid produced lower hardness values than those with peracetic acid. However, it was found that neither of the acids used differed from the control.

Hardness is the force required to achieve a certain deformation [48]. Contrary to the results found in this study, Gu et al. [49] analyzed the use of lactic acid on tilapia products (restructured fish and surimi products), where they observed an increase in hardness with a higher concentration of lactic acid in the solution. Bainy et al. [48] show texture is directly linked to pH. The lower the pH, the more rigid the meat is because acidic conditions result in the denaturation of proteins. This decreases the water-retention capacity and results in stiffer meat. Our study did not observe such behavior, as the treatments performed by immersion, regardless of the acid used, did not differ from the control. This finding is in line with a study by Rebouças et al. [50], in which samples of tilapia grown in freshwater, which had a lower pH value, were characterized as softer meat when compared to tilapia that was raised in salt water, which had a higher pH.

Texture is an important indicator of quality in fish. Unlike poultry and mammals, where softer meat is preferred by consumers, in the case of fish fillets, the best quality is firm and cohesive meat with a good water retention capacity [51]. Therefore, immersion in peracetic acid improved the quality of the tilapia fillets, as the hardness was higher than that in the other treatments. A similar result was found by Monirul et al. [31], where fish fillets (*Hypophthalmichthys molitrix*) that received a spray application of 1% acetic acid followed by treatment with 2% ascorbic acid, which reduced the growth and activity of microorganisms, resulted in fillets with a firmer texture and an extended shelf-life of up to 9 days.

The shear force measurements presented values between 1.31 and 1.61 kgf/cm^2^, with an average of 1.43 kgf/cm^2^, similar to the value of 1.45 kgf/cm^2^ found by Rebouças et al. [50] for freshwater tilapia meat. No significant effect between treatments or applied acids was noted for the other texture variables.

In addition to the effect on the quality parameters analyzed in this study, other criteria can also be used to investigate the impact of organic acids on the quality of fish, such as compounds from lipid oxidation and changes in sensory attributes. Mohan, Ravishankar et al. [52] observed an acceptability of up to 21 days for fish steaks (*Scomberomorus commerson*) immersed in sodium acetate (2%), while untreated steaks had an acceptability of 12 days. Furthermore, the authors showed that lipid oxidation, as measured by the thiobarbituric acid (TBA) index, was significantly reduced in samples treated with sodium acetate. In another experiment, Ahmed et al. [53] observed a reduction in TVB-N, peroxide, and TBA in fish (*Lates calcarifer*) immersed (60 s) in acetic acid (1%) and chitosan (1% and 2%) solutions, when compared to control samples.

The measurement of TVB-N indicates the freshness of fish. It consists of the quantification of low-molecular-weight compounds, such as trimethylamine (TMA), dimethylamine (DMA), and ammonia, which are formed during the fish spoilage process [54]. In countries such as Brazil, the maximum amount of TVB-N allowed in fish, according to the Ministry of Agriculture, Livestock, and Supply, is 30 mg N/100 g of fish for different species of fish, except species from the families *Salmonidae*, *Gadidae,* and *Merluccidae,* which must have a maximum of 35 mg N/100 g of fish. Elasmobranchs can have a maximum of 40 mg N/100 g of muscle tissue [44].

**Figure 3:**
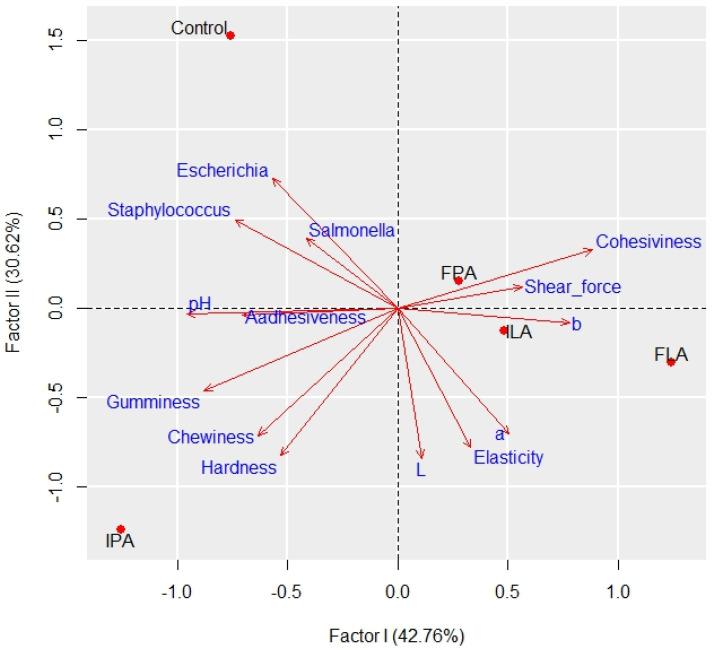
Multivariate factor analysis of the effect of acids and application methods on bacteria and the physicochemical qualities of the fillets. FLA (lactic acid fogging), ILA (lactic acid immersion), FPA (peracetic acid fogging), and IPA (peracetic acid immersion).

Multivariate factor analysis found that the first and second factors explained 73.38% of the total variation in the data, with the first being responsible for 42.76% and the second for 30.62% (Figure 3).

Only the first and second principal components have eigenvalues (variances) greater than 1. According to Mingoti [54], the number of components used to describe the total variation in the data must explain at least 70% and/or even the variance thereof must explain at least the average variation in the components, which is equal to 1 for standardized data.

The first principal component showed high negative correlations (r < −0.70) with pH, gumminess, *Staphylococcus aureus*, and adhesiveness and high positive correlations (r > 0.70) with cohesiveness and b. The second principal component showed high positive correlations with *Escherichia coli* and high negative correlations with L, a, hardness, chewiness, and elasticity. These correlations are evident from the positioning of the arrows in the principal component analysis graphs, where variables more strongly associated with the first principal component show a greater shift along the horizontal axis. In comparison, those more strongly associated with the second component show a greater shift along the vertical axis. Consequently, variables with high correlations in the same direction within the same component are directly related. In contrast, those with high correlations in the opposite direction of the component are inversely related.

Through factor analysis, it was possible to graphically verify that the analyzed variables were able to discriminate the treatments coherently, complementing the univariate analysis; furthermore, it was observed that the treatments using immersion in peracetic acid and the control were more similar to each other, different from the treatments with lactic acid and fogging with peracetic acid.

Thus, both the results of this research and those found in the literature show that using organic acids by different methods can be an additional technology for conventional fish conservation techniques. Furthermore, it can be seen in Figure 2 that both systems (fogging and immersion) can be applied on an industrial scale, reinforcing the robustness and practical applicability of the present study. Additionally, for commercial implementation, future research should evaluate different processing conditions (time, concentration, application method, and the type of organic acid), not only for the question of antimicrobial effects but also concerning the effects on physical and chemical characteristics, along with the sensory attributes of the fish. Another area that should be evaluated is the impact of acid use on the shelf-life of these fish products.

## 4. Conclusions

The results of this study indicate that fogging and immersion are alternatives for reducing *S.* Typhimurium, *E. coli*, and *S. aureus* in tilapia fillets and that these methods can positively affect fillet color. However, fogging, regardless of the acid used, was the best method for controlling *S.* Typhimurium and *S. aureus*, with results equal to immersion for *E. coli*.

This is the first study to investigate using organic acids (lactic and peracetic acid), applied by fogging, on tilapia fillets. The results demonstrate that the fogging of lactic and peracetic acid can be used as a decontamination system for tilapia fillets when applied near the end of the production line. In conclusion, fogging these acids can reduce microbial contamination in fish and limit sanitizer consumption.

## Figures and Tables

**Table 1:** Reduction in the population of the pool of *S*. Typhimurium, *E. coli*, and *S. aureus* in tilapia fillets after the application of lactic acid (3%) and peracetic acid (300 ppm) by fogging (15 min) or immersion (2 s).

Acids	Treatment	*S.* Typhimurium (log_10_ CFU/g)	*E. coli* (log_10_ CFU/g)	*S. aureus* (log_10_ CFU/g)
Control		6.78 ± 0.33	6.36 ± 0.32	6.56 ± 0.35
Peracetic	Immersion	6.03 ± 0.60 ^a^	5.25 ± 0.67 *	5.96 ± 0.64 ^a^
Peracetic	Fogging	5.12 ± 0.72 ^b^*	4.92 ± 0.46 *	5.58 ± 0.91 ^b^*
Lactic	Immersion	6.58 ± 0.2 ^a^	5.33 ± 0.39 *	5.05 ± 0.40 ^b^*
Lactic	Fogging	5.55 ± 0.37 ^b^*	4.87 ± 0.44 *	5.39 ± 0.55 ^b^*

Note: Values are means ± standard error. Means with different letters in the same column indicate significant differences (*p* ≤ 0.05) between treatments by the Scott–Knott test. * indicates a significant difference (*p* ≤ 0.05) by Dunnett’s test from the control treatment, comparing the results in the same column.

**Table 2:** Colorimetric parameters and pH values of tilapia fillets subjected to lactic acid (3%) and peracetic acid (300 ppm) treatments by means of fogging (15 min) and immersion (2 s).

Acids	Treatment	L*	a*	b*	pH
Control		46.44 ± 0.37	−4.13 ± 0.07	1.88 ± 0.10	6.58 ± 0.03
Peracetic	Immersion	52.33 ± 0.24 ^a^*	−3.59 ± 0.04 *	1.75 ± 0.08 ^b^	6.69 ± 0.06 ^a^
Peracetic	Fogging	47.70 ± 0.21 ^c^*	−3.38 ± 0.08 *	1.75 ± 0.54 ^b^	6.38 ± 0.02 ^b^
Lactic	Immersion	52.45 ± 0.40 ^a^*	−3.52 ± 0.15 *	2.45 ± 0.29 ^a^	6.25 ± 0.04 ^b^
Lactic	Fogging	50.69 ± 0.26 ^b^*	−3.52 ±0.06 *	2.51 ± 0.19 ^a^	6.26 ± 0.04 ^b^

Note: Values are means ± standard error. Means with different letters in the same column indicate significant differences (*p* ≤ 0.05) between treatments by the Scott–Knott test. * indicates a significant difference (*p* ≤ 0.05) by Dunnett’s test from the control treatment, comparing the results in the same column. L*: brightness (brightness, 0 = black and 100 = white), a*: redness (positive values = red and negative values = green), b*: yellow (positive values = yellow and negative values = blue).

**Table 3:** Texture profile and shear force of tilapia fillets after treatments with lactic acid (3%) or peracetic acid (300 ppm) by means of fogging (15 min) and immersion (2 s).

Acids	Treatment	Hardness (N)	Adhesiveness (mJ)	Gumminess (N)	Chewiness (mJ)	Cohesiveness	Elasticity (mm)	Shear Force (kgf/cm^2^)
Control		17.21 ± 0.24	0.53 ± 0.20	12.55 ± 2.52	33.90 ± 7.57	0.52 ± 0.06	2.68 ± 0.11	1.43 ± 0.39
Peracetic	Immersion	31.71 ± 1.38 ^a^	0.63 ± 0.21	18.52 ± 2.87	63.67 ± 11.61	0.41 ± 0.01	3.44 ± 0.28	1.35 ± 0.22
Peracetic	Fogging	20.22 ± 2.32 ^b^	0.50 ± 0.05	10.63 ± 1.79	29.33 ± 3.00	0.52 ± 0.02	2.82 ± 0.20	1.41 ± 0.12
Lactic	Immersion	19.10 ± 4.74 ^b^	0.63 ± 0.17	11.24 ± 2.35	37.30 ± 11.46	0.60 ± 0.02	3.17 ± 0.37	1.31 ± 0.13
Lactic	Fogging	20.68 ± 1.28 ^b^	0.17 ± 0.06	9.48 ± 2.25	36.06 ± 10.49	0.60 ± 0.03	3.66 ± 0.30	1.61 ± 0.19

Note: Values are means ± standard error. Means with different letters in the same column indicate significant differences (*p* ≤ 0.05) between treatments by the Scott–Knott test.

## Data Availability

The original contributions presented in the study are included in the article, further inquiries can be directed to the corresponding author.
